# Combined Analysis of Gut Microbiota and Plasma Metabolites Reveals the Effect of Red-Fleshed Apple Anthocyanin Extract on Dysfunction of Mice Reproductive System Induced by Busulfan

**DOI:** 10.3389/fnut.2021.802352

**Published:** 2022-01-12

**Authors:** Bin Wang, Jihua Xu, Shenhui Jiang, Yanbo Wang, Jun Zhu, Yugang Zhang

**Affiliations:** ^1^Engineering Laboratory of Genetic Improvement of Horticultural Crops of Shandong Province, Qingdao Agricultural University, Qingdao, China; ^2^College of Horticulture, Qingdao Agricultural University, Qingdao, China; ^3^College of Life Sciences, Qingdao Agricultural University, Qingdao, China

**Keywords:** red-fleshed apple, busulfan, spermatogenesis, metabolome, microbiota

## Abstract

Busulfan is currently an indispensable anti-cancer drug, but the side effects on male reproductive system are so serious. Meanwhile, red-fleshed apples are natural products with high anthocyanin content. In this research, we analyzed the effect of red-fleshed apple anthocyanin extract (RAAE) on busulfan-treated mice. Compared with the busulfan group, main plasma biochemical indicators were significantly improved after RAAE treatment. Compared with BA0 (busulfan without RAAE) group, total antioxidant capacity(T-AOC) and the activity of superoxide dismutase (SOD) and glutathione catalase (GSH-Px) in RAAE treatment groups were obviously increased, while the activity of alanine aminotransferase (ALT) and aspartate aminotransferase (AST) were significantly decreased. Malondialdehyde (MDA) was significantly decreased in the RAAE groups. In addition, we found RAAE alleviated busulfan-disrupted spermatogenesis through improving genes expression which are important for spermatogenesis, such as *DDX4, PGK2*, and *TP1*. Furthermore, we found that RAAE increased beneficial bacteria Akkermansia and Lactobacillaceae, and significantly depleted harmful bacteria Erysipelotrichia. The correlation studies indicated that RAAE ameliorated busulfan-induced rise in LysoPC levels through regulating gut microbial community and their associated metabolites. In conclusion, this study extends our understanding of the alleviated effect of RAAE on busulfan-induced male reproductive dysfunction through regulating the relationships between gut microbiota and metabolites.

## Introduction

Red-fleshed apple is a type of valuable and special variety of fruit and has always been attractive to consumers due to the red flesh color. Red-fleshed apple is rich in polysaccharides, anthocyanins and other flavonoids, and its flavonoids content is several times more than ordinary white apple (1). Anthocyanins can effectively inhibit oxygen free radicals and maintain the balance of the radicals (2). Thus, it exerts antioxidant effects in the body, having significant therapeutic effects on many chronic diseases (2). Study found the anthocyanin extracts of red-fleshed apple “Bekran” are effective in scavenging reactive oxygen species such as peroxides and superoxide anions (3). Studies showed that RAAE could significantly decreased the liver malondialdehyde activity and prevent the CCl_4_-induced decrease in liver superoxide dismutase, glutathione peroxidase, catalase, and reduce glutathione levels (4). Thus, Red-fleshed apple is a natural antioxidant with significant antioxidative activities and liver protective effects. Study found that among the four species studied, “Roberts Crab” had the highest content of total phenols, flavonoids and anthocyanins, and the strongest scavenging ability on DPPH, ABTS, and FRAP (5).

It has been demonstrated that anthocyanins are actually complex mixtures of flavonoids which have multiple effects on human health maintenance. Anthocyanins are highly hydrophilic, which makes it play a protective role in various pathophysiological conditions, such as improving subjective symptoms of visual fatigue after the task (6), preventing of cardiovascular diseases (7), and protecting of the liver from damage (8). The anthocyanin-rich lingonberry extracts blocked signal transducer and activator of transcription 3 (STAT3) and inhibited NF-κB expression (9). In addition, anthocyanin administration promoted the recovery of metabolites involved in glycerophospholipid metabolism, insulin signaling pathway and glutathione metabolism in the livers of obese mice, and study suggested that anthocyanin may ameliorate diet-induced obesity by alleviating oxidative stress and regulating lipid metabolism (2). Compared with traditional antioxidants, the extraction of anthocyanin from natural products has many advantages, including safety, accessibility and low cost (10). Therefore, it is of great significance to focus on the research and development of natural anthocyanin products.

Busulfan, also known as Myleran, is a representative drug of the methanesulfonate class. It was introduced in the middle of the 20th century for the treatment of chronic granulocytic leukemia and then gradually became the most widely used drug in the pretreatment of hematopoietic stem cell transplantation (11). However, with the widespread use of busulfan, its drawbacks are becoming clear, and one of the very serious side effects is its toxicity to male reproductive system (12). With the increase of the level of reactive oxygen species in the body, busulfan can destroy testicular germ cells, reduce testicular weight and sperm viability and increase the probability of sperm malformation and oligospermia, finally causing the transient or permanent infertility (13). After entering the cells, busulfan firstly consumes a large amount of glutathione (GSH) in the mitochondria. This results in a dramatic increase in oxidizing substances within the cell. The oxidative stress state of cells is activated and increased, which leads to the activation of relevant signaling pathways, and ultimately leads to cell apoptosis (14). Under these circumstances, we must take pharmacological measures in order to minimize its negative effects. It seems that the use of L-carnitine with busulfan decreases some side effects of this drug on the male reproductive system (15). There is a complex dynamic balance between intestinal microbe and host. Once the balance is broken, intestinal microbe structure will be disturbed. Intestinal microorganisms play an important role in vitamin synthesis, carbohydrate decomposition and other immune function metabolism, as well as *in vivo* and *in vitro* biological metabolism (16). Studies have shown that anthocyanins are rapidly absorbed by the stomach and small intestine through various mechanisms, and the unabsorbed anthocyanins are widely metabolized by the intestinal flora to produce a large number of anthocyanin metabolites and catabolites that are excreted circularly (17). Anthocyanins can promote the growth of beneficial bacteria lactobacillus and Bifidobacterium, while reducing potentially harmful bacteria such as Clostridium histolytic, which is associated with pro-tumor properties and inflammatory bowel disease (18). In recent years, some natural products have attracted much attention for their excellent antioxidant capacity, such as alginate oligosaccharides, tea polyphenols and melatonin. Korean red ginseng was found could attenuate the disruption of spermatogenesis caused by busulfan (19), and it was also reported garlic extract ameliorated the negative effects of busulfan on spermatogenesis (20). In our previous studies, it was found that red-fleshed apples have significant antioxidant capacity *in vitro* (21, 22). Furthermore, we conducted *in vivo* study using mice as a model and found that Red-fleshed Apple Anthocyanin Extract (RAAE) treatments showed ameliorative effects on male reproductive system dysfunction caused by busulfan (23).

In this study, we further investigated the mechanism of ameliorative effect of RAAE on testicular injury *in vivo* induced by busulfan in mice. We found that anthocyanin could reduce the oxidative damage caused by busulfan in mice by reducing the content of ROS, and the restoration of the mice reproductive system was mainly achieved through the regulation of genes related to the spermatogenesis pathway. In addition, we also investigated the effect of RAAE on the improvement of blood metabolism and intestinal microorganisms in mice with reproductive injury. Our study indicated anthocyanin could regulate testicular spermatogenesis through interfering metabolites such as Asparaginyl-Isoleucine in the blood and microorganisms such as Lactobacillaceae in the intestine. This research provides a theoretical basis for the development and application of functional products from red-fleshed apples.

## Materials and Methods

### Anthocyanin Preparation

The preprocessing of red-fleshed apples and the preparation of RAAE was performed strictly as per the previous study (23). The obtained RAAE was stored at −80°C for later research.

### Animal Study

All animal experimental procedures were conducted according to the protocol of the Animal Care and Use Committee of Qingdao Agricultural University [license number: SYXK (SD) 20170005]. Specific Pathogen Free (SPG) grade male ICR mice (5 weeks old, weight 30.0 ± 2.0 g) were raised in SPF chambers at 22 ± 2°C, 12: 12 h light-dark cycle, and 50–70% humidity. After 3 days of adaptation to the living environment, all mice were randomly divided into five groups (10 in each group, with one mouse died in each of the BA1 and BA5 groups), namely A0 group, BA0 group, BA0.1 group, BA1 group, and BA5 group. The freshly prepared RAAE was originally concentrated at 3000 mg/L, which was diluted to different concentrations and then used to feed the mice. The control group (A0) received saline by oral gavage, and the pathological control group (BA0) was given saline and busulfan (40 mg/kg, intraperitoneal injection); the other three groups were given different concentrations of RAAE orally (0.1 mg/kg, BA0.1; 1 mg/kg, BA1; and 5 mg/kg, BA5). In addition, these three groups also received intraperitoneal injections of busulfan before RAAE treatment (40 mg/kg), the same as BA0. The volume of gavage was 0.1 mL/mouse for 5 weeks.

### Sample Collection

After 35 days, the mice were humanely euthanized by cervical dislocation. The tissues were immediately separated, weighted, and stored at −80°C for further analysis.

### Biochemical Assays of Mice Plasma and Testicular Tissue

Total antioxidant capacity (T-AOC), aspartate aminotransferase (AST), alanine aminotransferase (ALT), and superoxide dismutase (SOD) and glutathione peroxidase (GSH-Px) and Malondialdehyde (MDA) were measured using kits from Nanjing Jiancheng Institute of Biological Engineering according to the manufacturer's instructions. All samples in each group were measured.

### Detection of Protein Levels and Location in Testis by Immunofluorescent Staining

Sections were first blocked with normal goat serum in PBS, followed by incubation (1:150 in PBS-1% BSA) with primary antibodies at 4°C overnight. After a brief wash, sections were incubated with an Alexa 546-labeled goat anti-rabbit or donkey anti-goat secondary antibodies (1:100 in PBS; Beyotime Institute of Biotechnology, Shanghai, P.R. China) at room temperature for 30 min and then counterstained with 40,6-diamidino-2-phenylindole (DAPI). The stained sections were visualized using a Nikon Eclipse TE2000-U fluorescence microscope (Nikon, Inc., Melville, NY), and the captured fluorescent images were analyzed using MetaMorph software. A minimum of 1000 cells were counted for each section, and a minimum of two to three tissue sections per animal were analyzed. Three animals from the control or busulfan exposed groups were analyzed. The number of positive cells was expressed as the percentage of total cells counted. The data was expressed as the average ± SD, N > 3.

### Western Blotting

Total proteins were extracted with RIPA lysate (Beyotime, Beijing, China) and incubated for 50 min at 100 V and 3.5 h at 120 V by SDS-PAGE using 4% stacking gel and 10% separating gel. The target proteins were further incubated with first antibody and diluted in buffer overnight at 4°C. The next morning, goat anti-rabbit-mouse or rabbit lgG secondary antibodies (Beyotime, Beijing, China) was conjugated with peroxidase (HRP) and incubated at room temperature. Pictures were obtained under a ECL (Sparkjade ECL plus, Shandong Sparkjade Biotechnology Co., Ltd.) detection system.

### Sequencing of Microbiota From Small Intestine Digesta Samples and Data Analysis

#### Genomics DNA Extraction

The microbial community DNA was extracted using MagPure Stool DNA KF kit B (Magen, China) following the manufacturer's instructions. DNA was quantified with a Qubit Fluorometer by using Qubit dsDNA BR Assay kit (Invitrogen, USA) and the quality was checked by running aliquot on 1% agarose gel.

#### Library Construction

Variable regions V3-V4 of bacterial 16S rRNA gene was amplified with degenerate PCR primers, 341F (5′-ACTCCTACGGGAGGCAGCAG-3′). Both forward and reverse primers were tagged with lllumina adapter, pad and linker sequences. PCR enrichment was performed in a 50 μL reaction containing 30 ng template, fusion PCR primer and PCR master mix. PCR cycling conditions were as follows: 94°C for 3 min, 30 cycles of 94°C for 30 s, 56°C for 45 s, 72°C for 45 s, and final extension for 10 min at 72°C for 10 min. The PCR products were purified with AmpureXP beads and eluted in Elution buffer. Libraries were qualified by the Agilent 2100 bioanalyzer (Agilent, USA). The validated libraries were used for sequencing on lllumina MiSeq platform (BGI, Shenzhen, China) following the standard pipelines of lllumina, and generating 2 × 300 bp paired-end reads.

#### Information Analysis Process

Filter the off-machine data, and use high-quality Clean data for post-analysis. Splice the reads into Tags through the overlap relationship between the reads. Cluster Tags into OTU, compare with database, and annotate species. Species complexity analysis, species difference analysis between groups, association analysis and model prediction were performed based on OTU and annotation results.

#### Filter Data

The original sequencing data is processed by the method of removing low quality according to the window: set a 25 bp window, if the average quality value of the window is <20, the back-end bases will be truncated from the window; the read length after truncation is lower than the original read 75% of the length, remove the entire sequence. Remove reads contaminated by connectors, reads containing N and low-complexity reads.

#### Connect to Tags

The software FLASH (Fast Length Adjustment of Short reads, v1.2.11) is used for sequence splicing, and the paired reads obtained by pair-end sequencing are assembled into a sequence by using the overlap relationship to obtain the tags of the hypervariable region. The minimum matching length is 15 bp, and the allowable mismatch rate in the overlapping region is 0.1.

#### OTU Clustering Result Statistics

Use the software USEARCH (v7.0.1090) to cluster the spliced Tags into OTUs. (1) Use UPARSE to cluster at 97% similarity to obtain the representative sequence of OTU; (2) Use UCHIME (v4.2.40) to remove the chimera generated by PCR amplification from the OTU representative sequence; (take the method of comparing with the existing chimera database gold database (v20110519) to remove the chimera.); (3) Use the usearch_global method to align all the tags back to the OTU representative sequence, and obtain the OTU abundance statistics table for each sample.

#### Linear Discriminant Analysis Effect Size Data Analysis

LEfSe determines the features (organisms, clades, operational taxonomic units, genes, or functions) most likely to explain differences between classes by coupling standard tests for statistical significance with additional tests encoding biological consistency and effect relevance (24). Threshold on the logarithmic LDA score for discriminative features was 1.5. Alpha value for the Kruskal-Wallis test among classes was 0.05. Pairwise comparisons among subclasses were performed not only among the subclasses with the same name. The strategy for multi-class analysis used more strict mode. Default structural parameters of the cladogram were used.

### Plasma Metabolite Measurements by LC-MS/MS

Plasma samples were collected and immediately stored at −80°C. Before LC-MS/MS analysis, the samples were thawed on ice and processed to remove proteins. Then samples were detected by ACQUITY UPLC and AB Sciex Triple TOF 5600 (LC/MS) as reported previously. 9–10 samples/group were analyzed for plasma.

### Analysis of Association Between Microbial Group (16S rRNA Sequencing) and Metabolic Group

After obtaining two sets of data, metabolites are pretreated based on identification information, and the relative abundance of microbes at each level in each sample is calculated based on absolute abundance. Then the association analysis of two groups was carried out based on metabolite abundance and microbial relative abundance.

### Statistical Analysis

The results were expressed as mean ± SD (standard deviation) from the triplicate independent measurements and analyzed using homogeneity of variance and one-way analysis of variance (ANOVA). In the Sequencing of microbiota from small intestine digesta samples, the rank sum test is used for data analysis, and the Wilcoxon Rank Sum Test is used for comparison of two samples. Use the Kruskal-Wallis Rank Sum Test when the sample content exceeds two and reaches three or more. Significant difference between animal groups was performed by least significant difference (LSD) test using SPSS 16.0 software (SPSS Inc., Chicago, IL). Significant differences were decided at *p* < 0.05.

### Data and Materials Availability

The microbiota raw sequencing data generated in this study has been uploaded to the NCBI SRA database with the accession number PRJNA 783722.

## Results

### RAAE Modifies Biochemical Parameters in Mice

Our previous work indicated the recovery effect of RAAE on seminiferous tubules damage caused by busulfan through histological and TUNEL analysis (23). In BA0 group, the number of spermatogonia, spermatocytes, leyding cell, and the diameter of seminiferous tubules was obviously reduced compared with control. RAAE treatment could recover the damage caused by busulfan especially for BA0.1 and BA 1 treatment. In addition, the alleviation effect of RAAE on busulfan induced liver damage was observed in our previous study (23). It has been reported that the organ damage induced by busulfan is caused by a large increase in the content of reactive oxygen species (ROS) including free radicals, causing oxidative damage in the cell (16).

**Figure 1:**
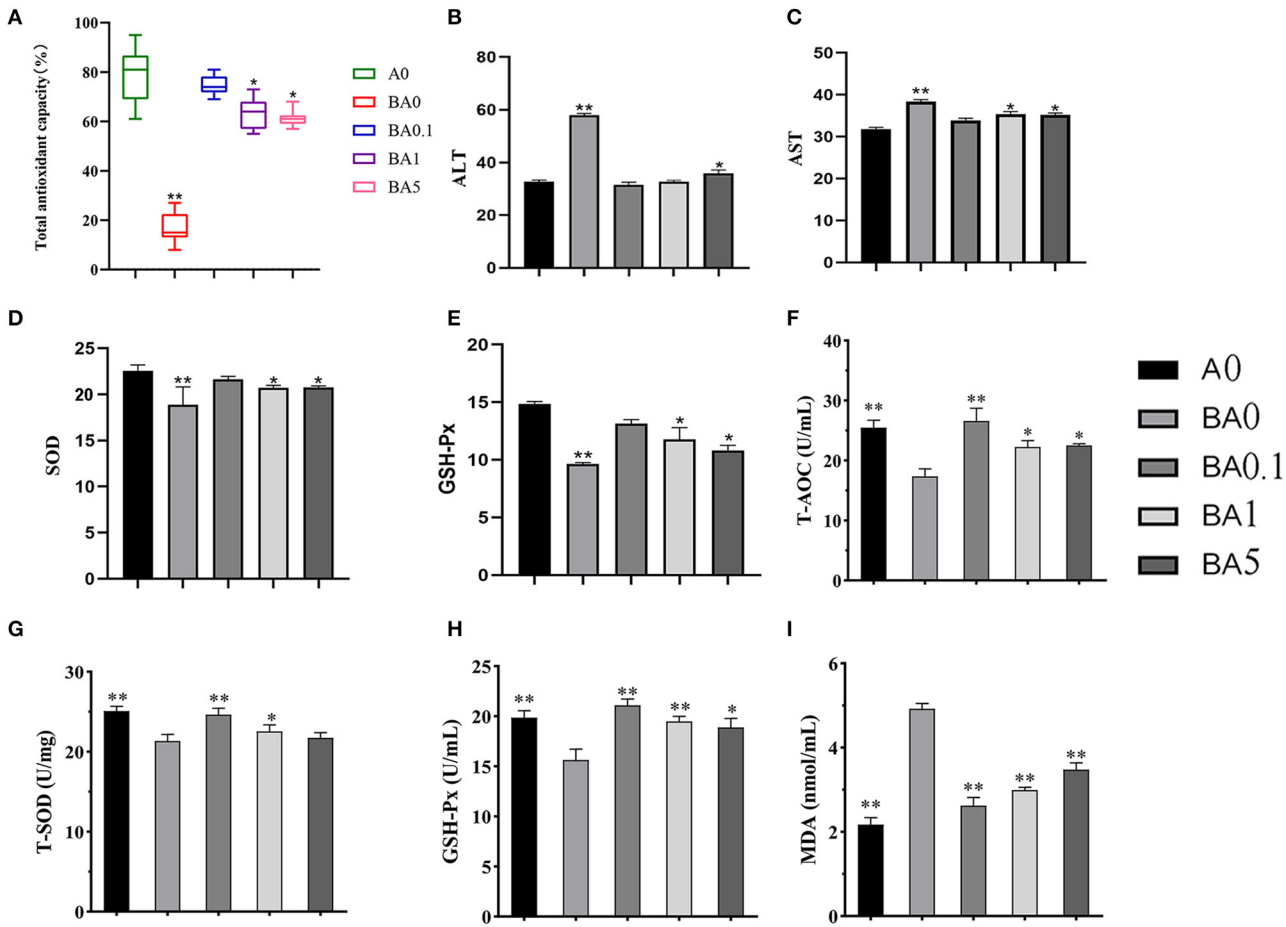
Effect of RAAE on mice of **(A)** Total antioxidant capacity (T-AOC) in plasm, **(B)** Aspartate aminotransferase (AST) in plasm, **(C)** Alanine aminotransferase (ALT) in plasm and **(D)** Superoxide dismutase (SOD) in plasm, **(E)** Glutathione peroxidase (GSH-Px) in plasm, **(F)** T-AOC in testis, **(G)** T-SOD in testis, **(H)** GSH-Px in testis, and **(I)** MDA in testis. Values are the means ± SD (*n* = 9 or 10). *Means 0.01 < *p* < 0.05 and **means *p* < 0.01.

Here, we detected the antioxidant activity and the related enzymes activity of plasma (Figure 1). The total antioxidant capacity (T-AOC) of the A0 group was 78.4%, while the BA0 group showed a highly decrease (Figure 1A). After intervention with anthocyanin extract, all three treatment groups showed significantly increased T-AOC levels, with BA0.1 restoring the T-AOC to 74.9%, which was very close to the T-AOC content of control group (Figure 1A). We further examined the levels of aspartate aminotransferase (ALT) and alanine aminotransferase (AST) in plasma (Figures 1B,C) and found that the ALT and AST levels in the mice of the BA0 group increased sharply, with an ALT activity of 57.7 U/L and an AST activity of 37.8 U/L, which also suggested the liver damage caused by busulfan administration. Whereas, BA0.1 reduced the ALT level to 30.8 U/L and the AST level to 33.2 U/L, which almost restored to normal levels as in A0. In addition, and the AST and ALT levels were also reduced in both BA1 and BA5 groups.

Antioxidant enzymes such as SOD and GSH-Px could protect the organisms against oxidative stress, and under normal conditions there is a physiological balance between ROS production and the antioxidant enzymes. Peroxidative damage in tissues often resulted in the depletion of antioxidant enzymes, such as SOD and GSH-Px. In this study, SOD and GSH-Px were 22.6 U/mL and 14.8 U/mL in the A0 group and decreased to 18.89 U/mL and 9.64 U/mL in the BA0 group, respectively. Compared with the BA0 group, the BA0.1, BA1, and BA5 groups all showed improvement, with the BA0.1 group showed the most effective improvement, with the content reaching 21.6 U/mL and 13.14 U/mL, respectively (Figures 1D,E).

Meanwhile, T-AOC, SOD and GSH-Px in testis showed the same trend as in plasma (Figures 1F–H). Compared with BA0 group, the total antioxidant capacity in testis increased significantly after RAAE intervention, almost the same as that in A0 group. Compared with BA0, SOD and GSH-Px indexes of testes in BA0.1 and BA1 groups were significantly increased, BA5 group also had a certain recovery effect. MDA in the detection index is the product of peroxidation of unsaturated fatty acids in biofilm destroyed by ROS. Further, we measured the MDA content in testis, because MDA content represents the level of lipid peroxidation reaction. The results showed that the biofilm of BA0 group was severely damaged, and MDA content was significantly higher than that of RAAE group (Figure 1I).

The recovery levels of SOD, GSH-Px and MDA indicated that the RAAE could improve the oxidative stress induced by busulfan, which helped to alleviate oxidative damage *in vivo*.

### RAAE Mitigation Effect of Busulfan-Induced Testicular Damage

**Figure 2:**
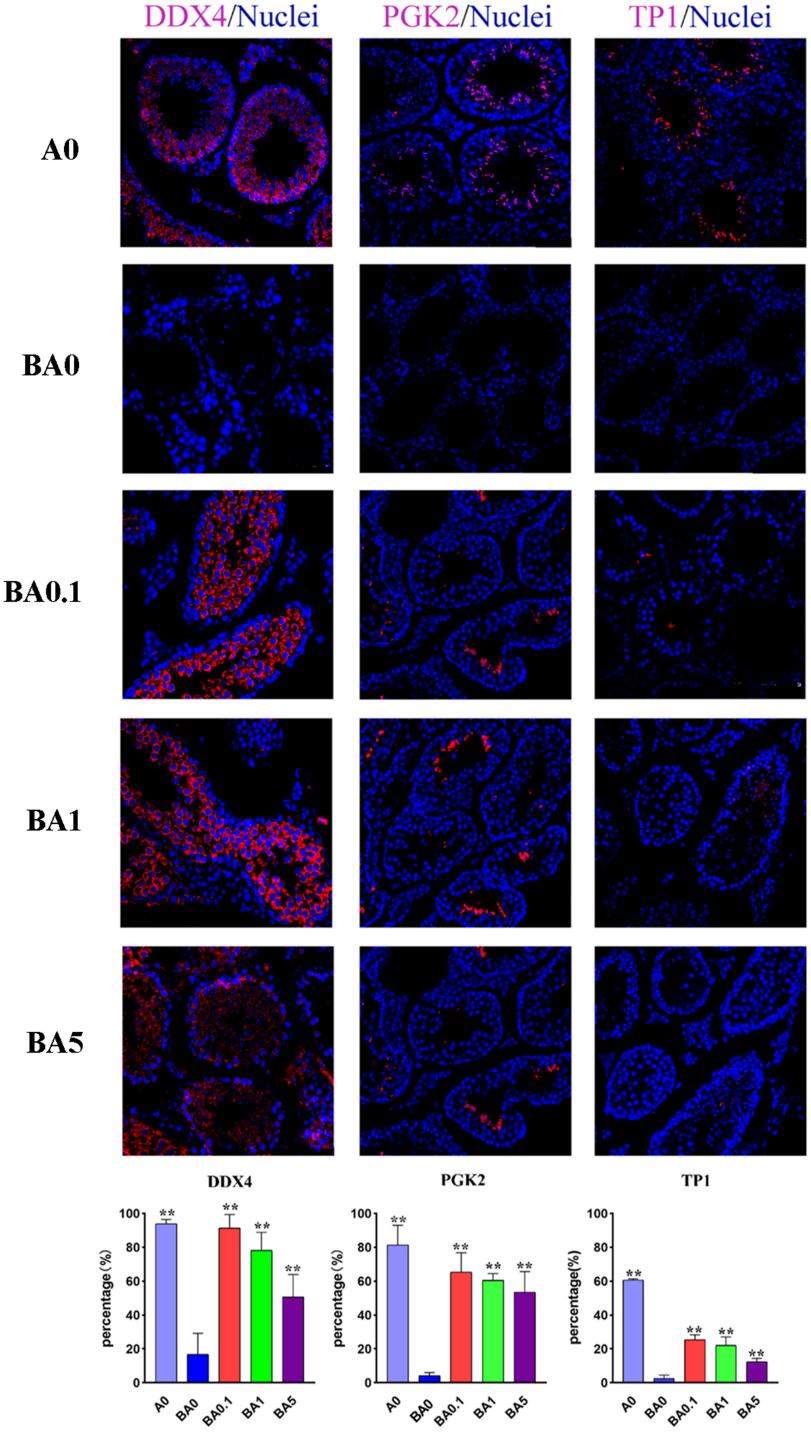
Immunohistochemical staining of the mice testis tissue in experimental groups; Light microscopy of mice testicular sections (×400 magnifications). Percentage of immunohistochemical positive cells in mouse testis. Values are the means ± SD (*n* = 9 or 10). **p < 0.01 vs. BA0.

Spermatogenesis is a complex process that is regulated by many genes. In males, *DDX4* is present in spermatocytes and round spermatids. In mice, *PGK2* is expressed in the pachytene spermatocyte stage. During the transformation of sperm cells, histones in the nucleus are converted into two transition proteins, called *TP1* and *TP2*. Disruption of the spermatogenesis process may result in a decrease in the number of protein-positive cells. In our study, the results of immunofluorescence staining showed that the percentage of *DDX4* positive cells in the A0 group was 93.93%, and the number in the BA0 group decreased sharply to only 16.73% (Figure 2). However, after RAAE treatment, the cell number in the three treatment groups increased significantly to 91.48, 78.21, and 50.68% in BA0.1, BA1, and BA5, respectively (*p* < 0.05). In particular, the percentage of positive cells in the BA0.1 group was similar to that in the A0 group. The percentage of *PGK2* positive cells in A0 group was 81.45%, and only 4.27% in the BA0 group. After the intervention of RAAE, the *PGK2* positive cells in BA0.1, BA1, and BA5 reached 65.36, 60.48, 53.58%, respectively, significantly higher than the level in BA0 (Figure 2). Similarly, compared with A0 group, the proportion of *TP1* positive cells in BA0 group decreased sharply. The recovery effect of *TP1* positive cells in BA0.1 group, BA1 group and BA5 group was obvious, and there was markedly different from that in BA0 group (*p* < 0.05) (Figure 2). In a word, the number of positive cells in the treated groups increased significantly after feeding RAAE, especially in the BA0.1 and BA1 group.

In order to verify the results, the apoptosis-related proteins *CYP11A1* and *CYP17*, antioxidant-related proteins *SOD1*, and spermatogenesis-related protein HSP70 were assayed in the testis tissue by Western blotting (Supplementary Figure S2). As shown, BA0 had an increased protein level of *CYP11A1* compared to A0, while it was decreased in the BA0.1 and BA1 group (Supplementary Figure S1), which indicated that RAAE had a positive effect on sperm maturation and Leydig cells growth. Similarly, *CYP17* was decreased in BA0.1, BA1, and BA5 group. Compared with the BA0 group, the abundance of *SOD1* was also increased in the BA0.1 group and BA1 group. Similarly, the level of *HSP70* in testis in BA0.1 was increased compared with BA0 (Supplementary Figure S1). These results suggested RAAE could improve the antioxidant capacity and decrease cell apoptosis to alleviate the damage induced by busulfan.

### Effects of RAAE Treatment on Composition and Relative Abundance of Gut Microbiota

**Figure 3:**
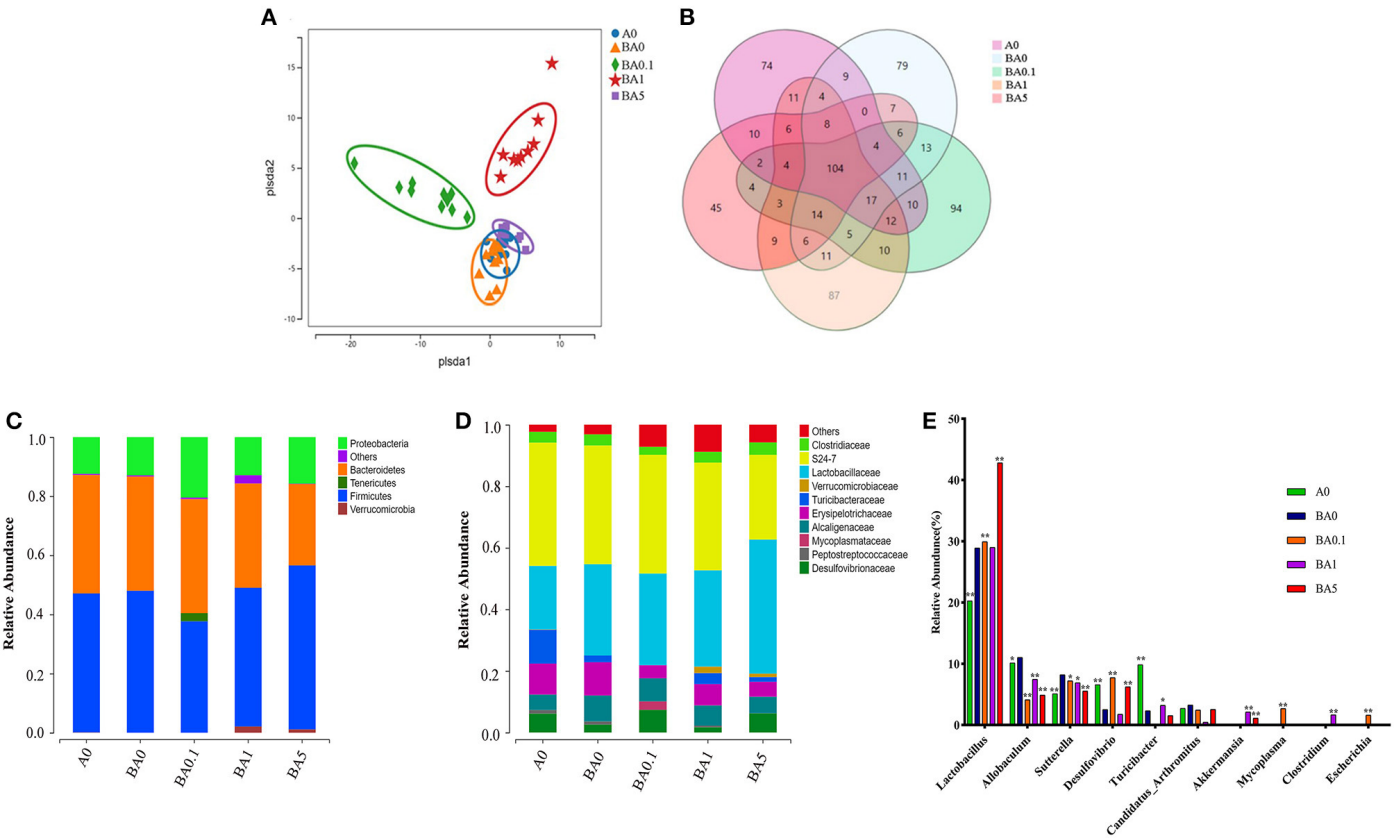
Effects of A on the taxonomic composition of the gut microbiota. **(A)** PLS-DA of the microbiota in different treatments, **(B)** The Venn of different groups, **(C)** Relative abundance of microbes at the phylum level, **(D)** Relative abundance of microbes at the family level, **(E)** Relative abundance of microbes at the genus level. Values are the means ± SD (*n* = 9 or 10). *Means 0.01 < *p* < 0.05 and **means *p* < 0.01 vs. BA0.

Earlier studies have reported that the gut microbiota is involved in the deconjugation and metabolism of testicular steroidogenesis (25). The gut microbiota affects the testis-pituitary axis, regulates lumen formation of the seminiferous tubules and the permeability of the blood-testis barrier (26). Due to the close association between intestinal microbes and male infertility, to investigate whether the rescue effect of RAAE on spermatogenesis is related to intestinal microbes, we sequenced the bacterial 16S rRNA V3-V4 region of the intestinal microbiota digestive system. Alpha diversity reflects the richness and diversity of microbial community within the group. The statistical results of Alpha index showed that Supplementary Figure S2, ACE index, Chao index, Shannon index and Simpson index had no significant difference among the five groups (*p* > 0.05). The Partial least squares discrimination analysis (PLS-DA) diagram illustrated that different treatment had variant effect on the abundance of intestinal flora in five groups (Figure 3A). BA0, BA0.1, BA1, and BA5 completely separated from each other, especially BA0.1 and BA1 which also distinguished themselves from BA0 group (Figure 3A). From Figure 3B, the total number of operational taxonomic unit (OTU) in the intestinal flora of the five groups of mice was 104, the number of OTU specific to A0 was 74, and the number of OTU specific to BA0, BA0.1, BA0.10, and BA5 groups were 79, 94, 87, and 45, respectively.

Interestingly, RAAE administration did not inhibit but showed an increasing trend toward the ratio of Firmicutes to Bacteroidetes (Figure 3C). Compared with the BA0 group, Verrucomicrobia content increased significantly in the BA1 and BA5 groups (*p* < 0.01). We further investigated the changes in gut microbial composition at the family level after busulfan treatment and RAAE intervention (Figure 3D). At the family level, in A0 group, S24-7 with an abundance of 40% was the predominant bacterial family, followed by Lactobacillaceae (20.63%), Turicibacteraceae (11.02%), Erysipelotrichaceae (10.02%). While in the BA0 group, S24-7 (38.56%) was the predominant bacterial family, followed by Lactobacillaceae (29.65%), Erysipelotrichaceae (10.80%), Alcaligenaceae (8.41%). These findings depicted that busulfan may increase the abundances of harmful bacteria, Erysipelotrichaceae and Alcaligenaceae. After 5-week RAAE intervention, the levels of Erysipelotrichaceae and Alcaligenaceae decreased significantly in BA0.1 group (*p* < 0.05). Compared with the BA0 group, the content of beneficial bacteria, such as Lactobacillaceae and Turicibacteraceae, increased markedly in some RAAE groups, for example, Lactobacillaceae increased from 29.65% in BA0 to 43.55% in BA5, Turicibacteraceae increased from 2.21% in BA0 to 3.59% in BA1, respectively. Meanwhile, the content of harmful bacteria Erysipelotrichaceae and Alcaligenaceae decreased in BA5 (10.8–4.84%, and 8.41–5.38%, respectively), Erysipelotrichaceae decreased from 10.80% in BA0 to 4.26% in BA0.1 (Figure 3D).

As shown in the Figure 3E, when comparing the differences among key intestinal microbes in all groups on the Genus level, it was found that beneficial bacteria Lactobacillus accounted for the largest proportion, up to 42.81% in the BA5 group, which is significantly increased compared with the A0 group (20.28%) and BA0 group (28.90%). Moreover, the content of harmful bacteria Allobaculum in BA0.1, BA1, and BA5 groups was 4.11, 7.47, and 4.89%, which was lower than that in BA0 group (11.01%). Compared with the BA0 group (8.19%), the proportion of harmful bacteria Sutterella in BA0.1 group, BA1 group and BA5 group decreased to 7.22, 6.89, and 5.54%, respectively (Figure 3E).

**Figure 4:**
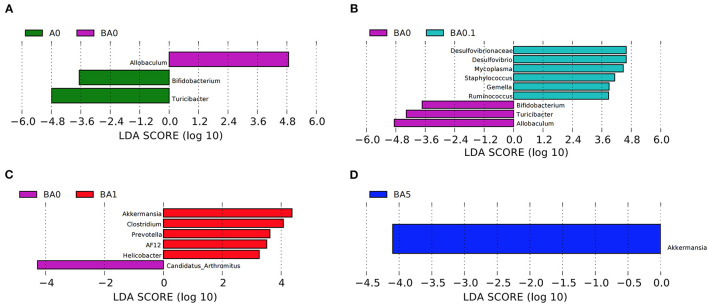
LEfSe was performed to determine the difference in abundance. **(A)**: A0 vs. BA0, **(B)**: BA0 vs. BA0.1, **(C)**: BA0 vs. BA1, **(D)**: BA0 vs. BA5 (n > 8 samples/group). The Biomaker with statistical difference was mainly displayed for species with significant difference LDA score greater than the pre-set value, and the default preset value was 2.0. The color of the bar chart represents each group, while the length represents the LDA score, which means the impact size of the species with significant differences between different groups.

Linear discriminant effect size (LEfSe) analysis showed that busulfan obviously impacted one bacterial genus on the basis of log_10_ LDA > 1.5 compared to the A0 group (*p* < 0.05, Figure 4A). The LEfSe analysis detected that six bacterial genera, such as Gemella (*p* < 0.05) and Ruminococcus (*p* < 0.01), increased significantly in BA0.1 group compared with those in BA0 group (Figure 4B). In the comparison between the BA0 group and the BA1 group, the intervention of RAAE increased beneficial bacteria, such as AF12and Akkermansia to improve the intestinal microenvironment (Figure 4C). The same result also detected in the comparison between BA5 group and BA0 group, the abundance of Akkermansia increased significantly in the BA5 group (*p* < 0.05, Figure 4D). The data revealed that the gut microbiota in busulfan mice was in a state of microbial dysregulation, and the RAAE treatment could neutralize the disorder of gut microbiota composition by increasing beneficial bacteria such as Akkermansia.

### Effects of RAAE on Plasma Metabolism

**Figure 5:**
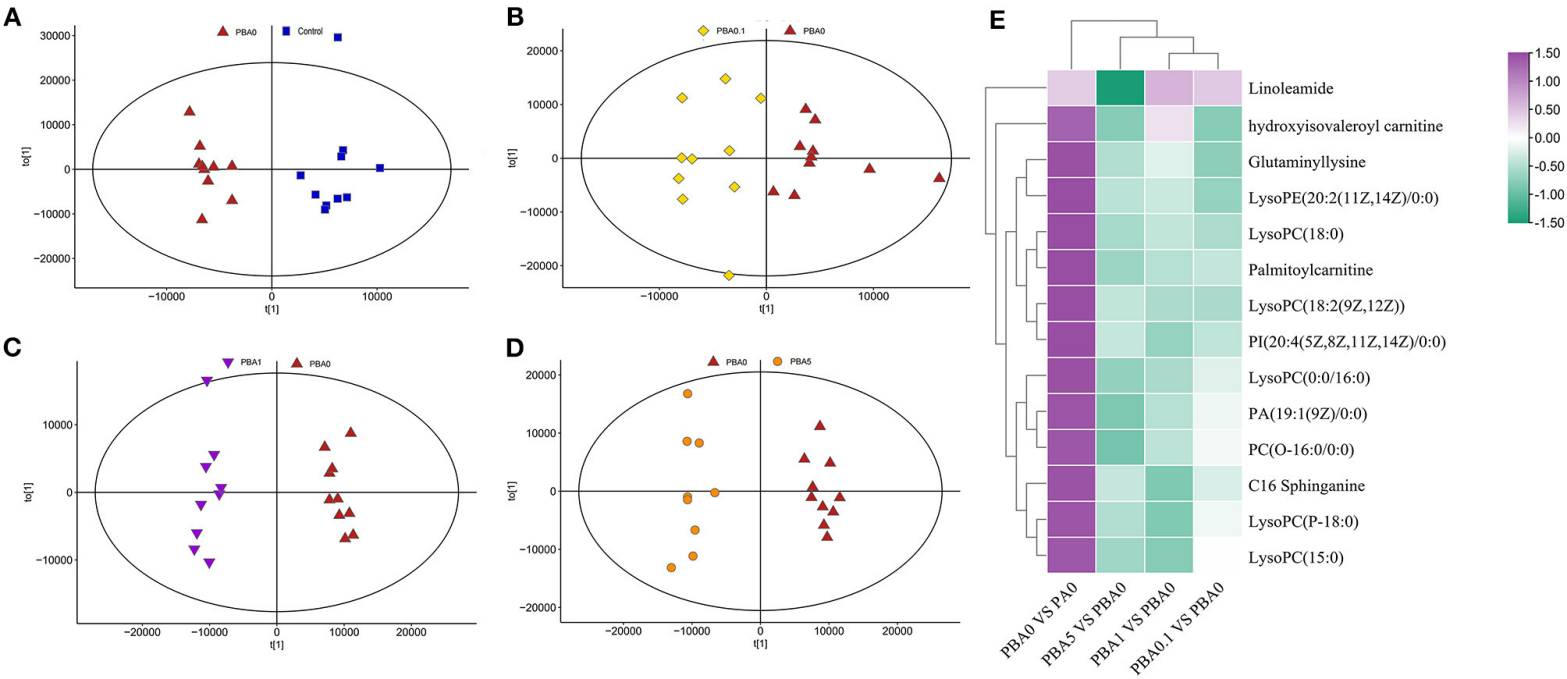
Plasma metabolome changes. **(A)** PLS-DA of mice plasm metabolites in the PBA0 and Control groups. **(B)** PLS-DA of mice plasm metabolites in the PBA0.1 and PBA0 groups. **(C)** PLS-DA of mice plasm metabolites in the PBA1 and PBA0 groups. **(D)** PLS-DA of mice plasm metabolites in the PBA5 and PBA0 groups. **(E)** Heatmap of changed plasm metabolites.

The plasma was analyzed in order to understand the metabolite changes *in vivo* after the RAAE intake in the mice. Data were analyzed by PLS-DA analysis, and PLS-DA score plots showed that the groups in the following pairings could be clearly separated: PBA0 and Control (Figure 5A), PBA0.1 and PBA0 (Figure 5B), PBA1 and PBA0 (Figure 5C), and PBA5 and PBA0 (Figure 5D). The data indicated a significant difference in the plasma metabolites between the five groups of mice, and busulfan changed the metabolic profiles in blood. Compared to PBA0, RAAE altered plasma metabolites. Fourteen significantly altered metabolites were simultaneously present in the four comparison groups, including PBA0 vs. PA0, PBA5 vs. PBA0, PBA1 vs. PBA0, and PBA0.1 vs. PBA0 (Figure 5E). Compared with the PA0 group, the levels of metabolites such as LysoPC and LysoPE were obviously increased in the PBA0 group and significantly decreased after RAAE treatment. The results indicated that the most changed metabolites were related to lipid metabolism, which is very important for spermatogenesis and male fertility.

### Effects of RAAE in Regulating the Correlation Between Gut Microbiota and Metabolites

**Figure 6:**
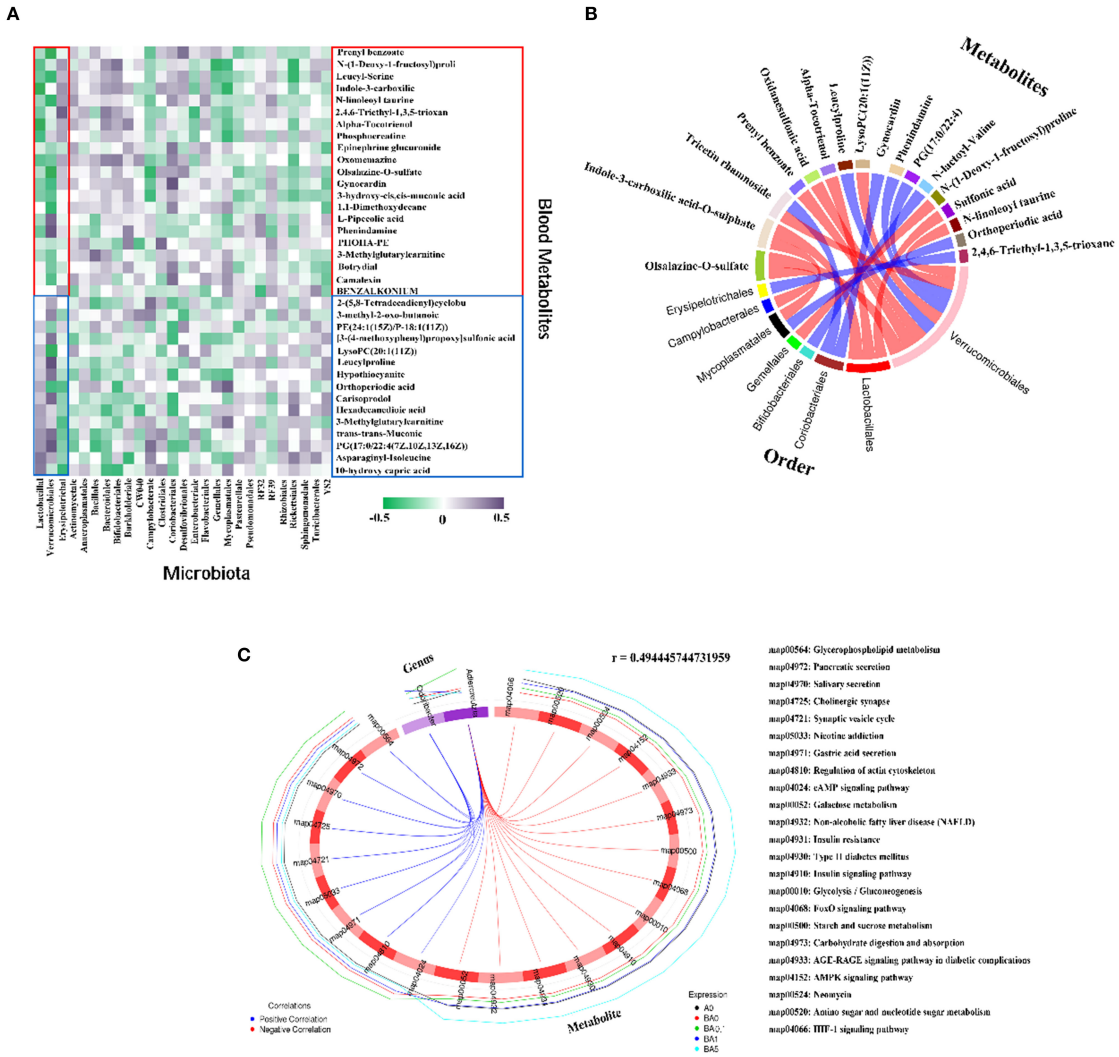
Correlation of changed intestinal microbiota and changed plasma metabolites. **(A)** Correlation between intestinal flora at order level and blood metabolites. **(B)** The correlation between each metabolite co-expressed cluster and each microbial group was analyzed by calculating the rank correlation coefficient, and the rank correlation was the strongest (the absolute value of the correlation coefficient was the largest and *P*-value < 0.05) of the first 20 relationship pairs are shown as chords. Each node represents a metabolite co-expression cluster or microbial group. The red arc between nodes represents negative correlation, while the blue arc represents positive correlation. **(C)** Correlation diagram of microbes at the Genus level and differential metabolic pathways. The broken line on the periphery of the ring represents the abundance value of the differential metabolite and the microbial taxa in each group, the distance between the broken lines represents the difference between the groups, the upper right corner is the correlation coefficient threshold, and only the differential metabolites with the absolute value of the correlation coefficient greater than the threshold. There is a connection with the microbial group. The blue connection represents a positive correlation, and the red connection represents a negative correlation.

Generally, gut microbiota metabolizes nutrients in the intestine and can also regulate intestinal metabolites to further influence the blood metabolism (25, 26). Therefore, the correlation coefficient between plasma metabolites and gut microbiota was analyzed. As shown in Figure 6A, 27 microbiotas, including Lactobacillal, Verrucomicrobiales, Erysipelotrichal, Actinomycetale, Anaeroplasmatales, Bacillales, Bacteroidales, Bifidobacteriales, Burkholderiales, CW040, Campylobacterales, Clostridiales, Coriobacteriales, Desulfovibrionales, Enterobacteriales, Flavobacteriales, Gemellales, Mycoplasmatales, Pasteurellales, Pseudomonadales, RF32, RF39, Rhizobiales, Rickettsiales, Sphingomonadales, Turicibacterales, YS2 had correlations with 36 plasma metabolites.

The beneficial microbe Lactobacillal and harmful microbe Erysipelotrichal were best correlated with these 36 metabolites (Figure 6A). Totally 21compounds of 36 plasma metabolites which were increased by busulfan (in A0 vs. BA0) and decreased by RAAE, were negatively correlated with Lactobacillal. Lactobacillal were more abundant in RAAE treatment groups (in BA0.1, BA1, and BA5) and less in A0 and BA0. The other 15 compounds, which were decreased by busulfan (in A0 vs. BA0) and increased by RAAE, were positively correlated with Lactobacillal. The correlation of Erysipelotrichal and blood metabolites was opposite to that between Lactobacillal and blood metabolites. Erysipelotrichal was positively correlated with the 21 compounds that were increased by busulfan and decreased by REEA. Moreover, Erysipelotrichal was less abundant in RAAE treatment groups. The data further indicated that these two dominant bacteria may be involved in RAAE modification of blood metabolites which may assist in the rescue of spermatogenesis.

In addition, the correlation between intestinal microbes and blood metabolites indicated that the beneficial microbe Lactobacillal help the recovery of blood metabolites, while harmful microbe Erysipelotrichal have a negative effect on blood metabolites (Figure 6A). Figure 6B shows a chord chart of the correlation between differential metabolites and microbiota in order level. The results further verified that Lactobacillal and Erysipelotrichal had strong correlation with various metabolites in plasma. In addition, we also found that Verrucomicrobiales has a strong correlation with a variety of metabolites and has a positive effect on metabolism. We further used Spearman's canonical correlation analysis to determine the association between the differential metabolic pathways and microbial groups in genus level (Figure 6C). And the first 23 pairs of relationships with the strongest typical correlation (the largest absolute value of the correlation coefficient) are displayed in the form of circular graphs. As beneficial intestinal microbial, Odoribacter and Adlercreuzia have positive correlation on nine metabolic pathways simultaneously. In addition, Adlercreuzia negatively regulated 14 metabolic pathways. The data further indicated that these bacteria may be involved in RAAE modification of blood metabolites, which may assist in the rescue of spermatogenesis.

**Figure 7:**
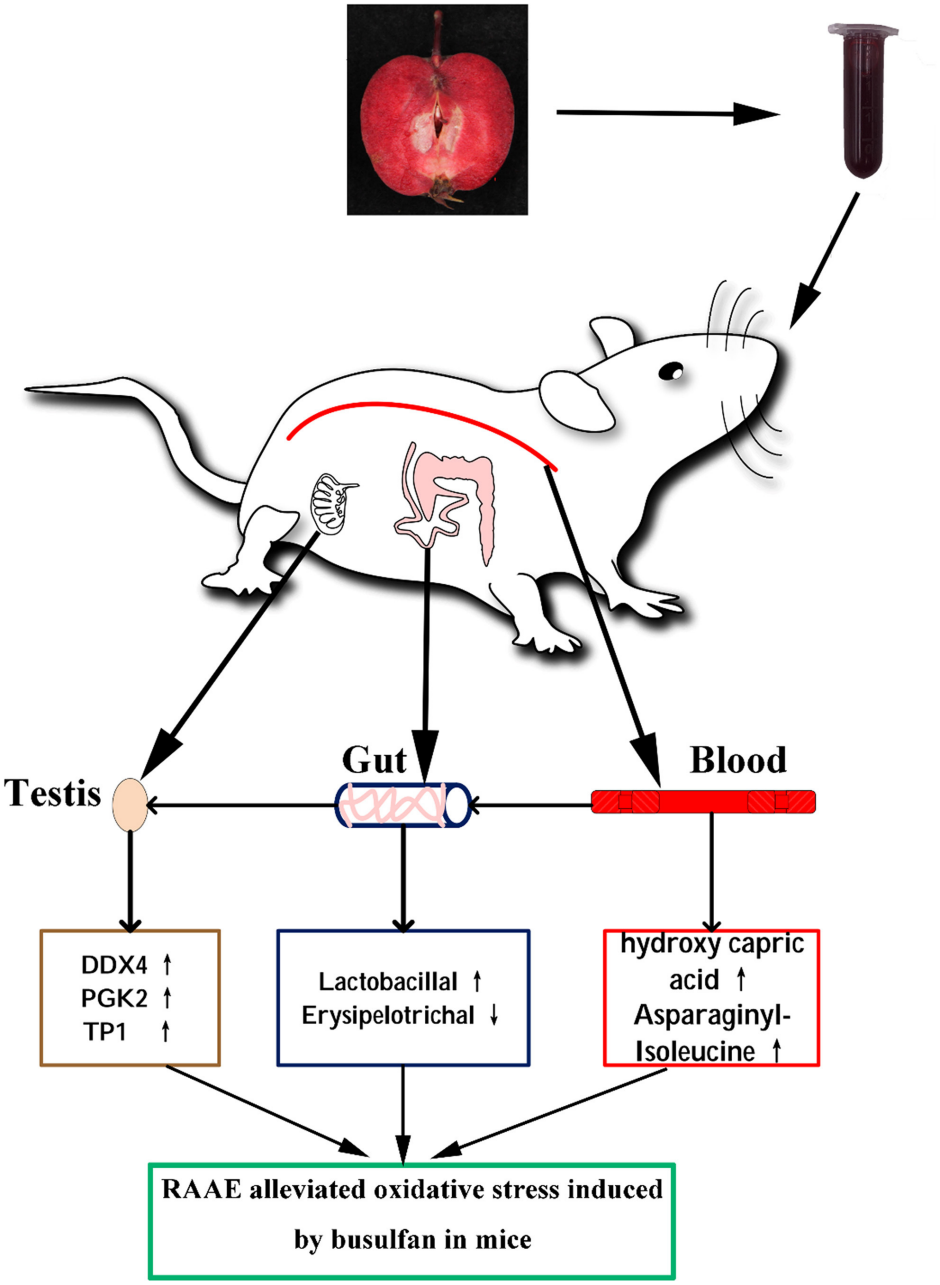
Proposed mechanism of RAAE alleviating oxidative stress induced by busulfan. The up arrow (↑) and down arrow (↓) represent up-regulating and downregulation effects of RAAE, respectively.

Taken together, RAAE may alleviate the destruction of busulfan-induced spermatogenesis by promoting germ cell development, blood metabolites and intestinal microorganisms (Figure 7).

## Discussion

It is well-known that the incidence of cancer has continued to rise in the past few decades. Chemotherapy along with alkylating agents such as busulfan is one of the effective treatments for leukemia. Busulfan is one of the very few anti-cancer drugs used in children under the age of three (27). However, the side effects of busulfan on the reproductive system was obvious. Busulfan can destroy testicular germ cells, decrease testis weight and sperm motility, increase sperm abnormalities and oligo-azoospermia rate, and finally cause temporary or permanent sterility (16).

Oxidative stress induced by busulfan may affect the unsaturated fatty acids in the sperm cell tail membrane, interfere with its fluidity, and reduce sperm motility (28). Anthocyanin compounds contain multiple phenolic hydroxyl donors in their molecules and are natural free radical scavengers. Research by Seeram proved that anthocyanin-lactoside can inhibit the lipid peroxidation induced by the reactive oxygen species (29). It was also reported that anthocyanin has anti-cancer effect because it could inhibit the proliferation and metastasis of cancer cells (30).

Our previous studies indicated RAAE had a strong ability to scavenge DPPH, superoxide anions and hydroxyl radicals, which further reduced the level of oxidative stress and exhibited stronger antioxidant capacity (21). Recently, our study showed that RAAE could significantly recover the seminiferous tubules damaged by busulfan, such as increasing the number of spermatogonia, spermatocyte and interstitial cells in testis, as well as improving the diameter of seminiferous tubules (23).

Increased content of glutathione in cells was detected as a result of rising level of reactive oxygen species caused by busulfan *in vivo*, which further affect the structure and physiological indexes of testis, resulting in male reproductive damage. In the present study, we examined the total antioxidant capacity (T-AOC) in the serum, which can directly reflect the level of reactive oxygen species *in vivo*. As shown in Figure 1A, the total antioxidant capacity was markedly increased in the anthocyanin-treated groups, especially in the BA0.1 group, which almost returned to normal levels. Therefore, it suggests that the RAAE used in this study has a significant therapeutic effect on the oxidative damage *in vivo* caused by busulfan.

Studies have reported the improvement effects of various plant extracts on busulfan-induced testicular toxicity (13, 16). In this study, we investigated the effect of different doses of RAAE (BA 0.1, BA1, and BA 5) on busulfan-treated animals. All anthocyanin supplementation can effectively reduce serum ALT and AST, increase plasma SOD, GSH-Px ability (Figures 1D,E), indicating the significant effect of anthocyanin on the scavenging ability of free radicals in plasma. T-AOC, SOD, and GSH-Px in testis showed the same trend as in plasma. MDA is a lipid peroxidation product in the body, and its level in the body represents the strength of lipid peroxidation reaction. These indicators reflect the antioxidant capacity of the body and are key indicators of oxidation and antioxidant defense system. The results showed that busulfan increased MDA content in testicular tissue and decreased T-AOC, SOD, and GSH-Px activities in testis and plasma, while the oxidation and antioxidant indexes in testicular tissue of RAAE treated mice had no significant difference compared with the A0 group. These results indicated that RAAE could alleviate the oxidative damage of testicular system induced by busulfan to a certain extent. However, BA5 (the highest dose) did not show a therapeutic effect proportional to its dose. We speculate that it may be due to the high concentration of anthocyanin. Similar results were reported in other natural extracts studies which showed that ultra-high doses of natural extracts did not show a dose-dependent effect on rat organ tissues, and may even have a negative effect on liver tissues (30).

Furthermore, as disruption of the spermatogenesis process by busulfan may result in a decrease in the number of protein-positive cells, and the beneficial effects of RAAE on male germ cell development were probably mainly due to the recovery of gene expression and protein levels of the prominent genes affecting spermatogenesis. To test this hypothesis, we selected the proteins for the marker genes [*DDX4, PGK2*, and *TP1* (10, 31, 32)] and determined the percentage of positive cells in the testis samples by immunofluorescence staining (Figure 2). The expression of *VASA* mRNA and protein was significantly decreased in the sperm of oligozoospermic men, which suggested the lower expression of the *VASA* gene might be associated with pathogenesis in some subtypes of male infertility and *VASA* could be used as a molecular marker for the diagnosis of male infertility (33). Transcription of the testis-specific *PGK2* gene is selectively activated in primary spermatocytes to provide a source of phosphoglycerate kinase that is critical to normal motility and fertility of mammalian spermatozoa (34). *TP1* is the spermatid (ST) marker gene (10) and is expressed in STs. In BA0 testicular tissue, three marker genes are involved in spermatogenesis, showed similar changes, suggesting that busulfan disrupted the process of spermatogenesis. However, it was increased in BA0.1 to almost similar levels as in A0. Studies have reported that Torsion-detorsion (T/D) led to a significant decrease in the number of both *DDX4* and P450-positive cells in the testis, whereas stromal vascular fraction (SVF) injection contributed to a significant increase in the number of both cells (35). And it has been shown that alginate oligosaccharides can alleviate the reproductive damage induced by busulfan by increasing the number of *TP1* marker genes. The data suggested that busulfan disrupted the process of spermatogenesis, while RAAE protected spermatogenesis by promoting the development of spermatogonia to spermatocytes and on to spermatids.

It is known that metabolic regulation is essential for spermatogenesis (36, 37). Many components such as hormones and other endogenous or exogenous factors have a synergistic contribution to the homeostasis of metabolism in the testis and the progression of spermatogenesis (37). In our previous study, we found that busulfan disrupted metabolism homeostasis in blood samples in mice, while RAAE reversed this change, such as downregulating lysophosphatidylcholine and upregulating L-arginine, glycine, and anandamide (23). Recently, there has been an increasing interest in the effects of the gut microbiota on human physiology. The intestinal microbiome is involved in the regulation of multiple host metabolic pathways, which not only plays a role in metabolism-related diseases, such as obesity and diabetes (25), but also affects other systems, such as the nervous system and reproductive system (38). Gut microbes help the digestive system function properly and maintain intestinal wall integrity. A healthy balanced gut flora both promotes the absorption of nutrients needed for healthy sperm formation, such as zinc, vitamin C, phosphorus, folic acid and magnesium, and prevents toxins and other harmful substances from crossing the intestinal barrier into the bloodstream. When intestinal permeability increases, harmful substances enter the bloodstream and put the immune system on high alert, leading to systemic inflammation, which may also cause oxidative stress to sperm, reducing sperm count and motility. It was found that algal oligosaccharides could increase the content of beneficial bacteria in intestinal tract and inhibit the content of harmful bacteria to regulate spermatogenesis (39). Gut microbiota from Alginate oligosaccharide dosed animals may improve spermatogenesis through benefit to the recipients gut microbes. Sperm quality and spermatogenesis were significantly improved after alginate oligosaccharide dose mice fecal microflora transplantation (40). It has been reported that gut microbiota can affect the reproductive performance of males and females and their offspring (25).

In our study, 16S rRNA analysis indicated that RAAE improved the relative abundance of intestinal microorganisms in mice induced by busulfan. Akkermansia, a dominant gut bacterium belongs to phylum Verrucomicrobia, has been reported to counteract the development of low-grade inflammation and attenuate gut barrier dysfunction (41). In our study, we observed that RAAE intervention dramatically enriched Akkermansia (*p* < 0.05). This is in line with the previous reports which demonstrated a higher Akkermansia abundance is associated with a healthier glucolipid metabolic status (41). Current studies have confirmed that Akkermansia is lower in several unhealthy conditions, such as obesity, diabetes, intestinal inflammation, liver disease, or chronic alcohol consumption (41). The intervention of RAAE significantly increased the content of Akkermansia bacteria in mice (*p* < 0.05). Intestinal epithelial cells are usually covered by a mucus layer (the main component is mucin). On the one hand, it can protect the intestinal epithelial cells from the invasion of microorganisms, and on the other hand, they can provide growth energy for microorganisms that use them as nutrients. Because Akkermansia has a special function of degrading mammalian intestinal mucin, its interaction with the body has attracted much attention. As a gut microbe, the relationship between the bacteria and host metabolism is not only reflected in the intake, utilization and consumption of energy related to glucose, protein and lipid metabolism, but also in the health of the mucous layer and mucosal immune response. The mechanism of potential microorganisms affecting host metabolism (42). With the development of research on the relationship between intestinal microbes and health, the relationship between Akkermansia and host health has also received high attention. Studies have found that its relative abundance is comparable to Inflammatory bowel disease, appendicitis (43), obesity (44, 45) and adolescent autism (46) and other diseases are negatively correlated. In addition, studies have found that it can also protect against atherosclerosis by improving the inflammatory response induced by endotoxemia in Apoe-/- mice (47). In summary, Akkermansia, as a kind of intestinal bacteria that can make good use of gastrointestinal mucin for growth, is closely related to immune response, lipid metabolism and other processes of the body, and plays an important role in maintaining body health. In addition, we also found that RAAE increased beneficial bacteria such as lactobacilli in the small intestine. Oral introduction may enhance non-specific host resistance to microbial pathogens and thereby facilitate the exclusion of pathogens in the gut (48).

The PLS-DA score plots revealed that indicated that both RAAE and busulfan changed the metabolic profiles in blood (Figure 5). From the analysis of plasma metabolism, we found that LysoPC was downregulated in PBA0.1, PBA1, and PBA5. Lipid metabolism has been suggested to play important roles in spermatogenesis and male fertility (39). Thus, the downregulation of LysoPC, which was observed in the RAAE treatment groups, could contribute to the reduction of damage caused by busulfan.

Intestinal microbes can metabolize nutrients in the gut and regulate intestinal microbes and influence blood metabolism (30). The abnormal metabolism of lipids in the reproductive system or blood contributes to male infertility in humans (49, 50). On the contrary, when passing through other organs, blood metabolites can affect their development and might cause disease. Our study suggests that under RAAE treatment, blood metabolites and gut microbiota interact to alleviate the damage during spermatogenesis caused by busulfan. The results of phylum analysis showed that RAAE had no expected decreasing trend in the proportion of Firmicutes and Bacteroidetes. However, RAAE significantly increased the content of Verrucomicrobia (*p* < 0.01). The harmful bacteria Erysipelotrichal are a type of dominant gut bacteria, which belongs to phylum Firmicutes. We found the RAAE significantly depleted the abundance of Erysipelotrichaceae (*p* < 0.05) in family level (Figure 3D). Lactobacillaceae are microorganisms that are beneficial to host health, fermenting sugars to produce lactic, maintaining health and regulating immune function. We found that the contents of Lactobacillaceae in BA5 group were significantly increased at family level and genus level (*p* < 0.01), thus protecting the host intestinal microenvironment. Furthermore, gut microbiota is responsible for metabolic functions (51), and found the correlation of gut microbiota and blood metabolites demonstrated that the “beneficial” microbe Lactobacillaceae assisted the recovery of blood metabolites (52) RAAE improved specific blood metabolites involved in small intestinal functions such as LysoPC, Prenyl benzoate and Alpha-Tocotrienol. It is worth noting that in previous metabonomics analysis, we found that RAAE treatment had a negative regulatory effect on LysoPC (20). LysoPC, resulted from the hydrolytic cleavage of phosphatidylcholine, is the primary component of low-density lipoprotein (53). LysoPC can bind to G protein-coupled receptors and Toll-like receptors to activate downstream signaling pathways, and play important biological roles in organisms such as increasing the pro-inflammatory cytokines, inducing oxidative stress, and producing apoptosis (53, 54). Thus, the down-regulation of LysoPC in our RAAE treatment groups could alleviate the damage caused by busulfan. In addition, Verrucomicrobiales was a negatively correlate with LysoPC, which could well reflect that RAAE regulates metabolism *in vivo* through intestinal colonies. In conclusion, the data confirm our hypothesis that RAAE has a profound effect on plasma metabolism and small intestinal function. RAAE-dosed mice were successful in improving small intestine function and blood metabolome. Our results suggest that RAAE could be used to prevent oxidative damage induced by chemotherapeutics or other factors.

In this study, we also analyzed the correlation between intestinal microbes and metabolic pathways. It turned out that at the genus level, Odoribacter and Adlercreuzia were significantly associated with 23 metabolic pathways. Odoribacter, another butyrate-producing bacterium, has been reported to be increased by pomegranate juice consumption in healthy subjects (55). Bacteria of the genus Adlercreutzia produce the metabolite equol, a known anti-inflammatory agent and vasodilator (56). Thus, it could be hypothesized that increases in populations of Adlercreutzia and its metabolites following RAAE administration might contribute to anti-inflammation. Both Odoribacter and Adlercreuzia had positive regulation effects on nine metabolic pathways, such as Pancreatic secretion, Cholinergic synapse. At the same time, Adlercreuzia negatively regulated 14 other metabolic pathways, such as Insulin resistance and Insulin signaling pathway. Gut microbiota can metabolize nutrients in the intestine and can also regulate intestinal metabolites to influence the blood metabolome.

Based on the study of biochemical indicators and immunofluorescent staining, our results demonstrated that taking RAAE daily for 5 weeks could alleviate testicular injury. Additionally, 16S rRNA high-throughput sequencing analysis demonstrated that RAAE could increase the relative abundance of intestinal flora (e.g., Akkermansia and Lactobacillus) in Busulfan-induced mice. Furthermore, the correlation analysis between plasma metabolites and intestinal flora showed that intestinal flora plays an important role in the regulation of plasma metabolites (e.g., LysoPC and LysoPE), thus alleviating the reproductive injury induced by busulfan. These results suggest that RAAE may be an effective candidate functional food.

## Data Availability Statement

The datasets presented in this study can be found in online repositories. The names of the repository/repositories and accession number(s) can be found at: https://www.ncbi.nlm.nih.gov/sra, PRJNA783722.

## Ethics Statement

The animal study was reviewed and approved by the Animal Care and Use Committee of Qingdao Agricultural University.

## Author Contributions

BW designed the research, performed the experiments, and wrote the paper. JX made contributions to perform the experiments and analysis the data. SJ provided experiments assistance to BW. YW provided tissue culture assistance to BW. JZ provided the suggestions and make contributions to design of the experiment. YZ conceived the original screening and plans and revise the manuscript. All authors contributed to the article and approved the submitted version.

## Funding

This work was financially supported by Natural Science Foundation of Shandong (ZR2019MC003), National Key R&D Program of China (2019YFD1001403), China Agriculture Research System Foundation (CARS-27), Qingdao Scientific Research Foundation (19-6-1-60-nsh), and the Qingdao Agricultural University Scientific Research Foundation (663/1120075).

## Conflict of Interest

The authors declare that the research was conducted in the absence of any commercial or financial relationships that could be construed as a potential conflict of interest.

## Publisher's Note

All claims expressed in this article are solely those of the authors and do not necessarily represent those of their affiliated organizations, or those of the publisher, the editors and the reviewers. Any product that may be evaluated in this article, or claim that may be made by its manufacturer, is not guaranteed or endorsed by the publisher.

## Supplementary Material

Supplementary Figure S1 **(A)** Protein levels of *CYP11A1, CYP17, SOD1*, and HSP70 in mice testis tissues detected by Western blotting, **(B)** Protein expression levels of *CYP11A1*, **(C)** Protein expression levels of *CYP17*, **(D)** Protein expression levels of *SOD1*, **(E)** Protein expression levels of *HSP70*, Relative expression was calculated using target protein/Actin. ^*^Means 0.01 < *p* < 0.05 and ^**^means *p* < 0.01.

Supplementary Figure S2 The alpha index of the small intestine microbiota: **(A)** ACE index, **(B)** Chao index, **(C)** Shannon index, **(D)** Simpson index (n > 8 sample/group).
