# Supplementary figures and images for: Combined Analysis of Gut Microbiota and Plasma Metabolites Reveals the Effect of Red-Fleshed Apple Anthocyanin Extract on Dysfunction of Mice Reproductive System Induced by Busulfan
Source: Front Nutr. 2022 Jan 12;8:802352. doi: 10.3389/fnut.2021.802352 (PMC8789878; doi:10.3389/fnut.2021.802352)

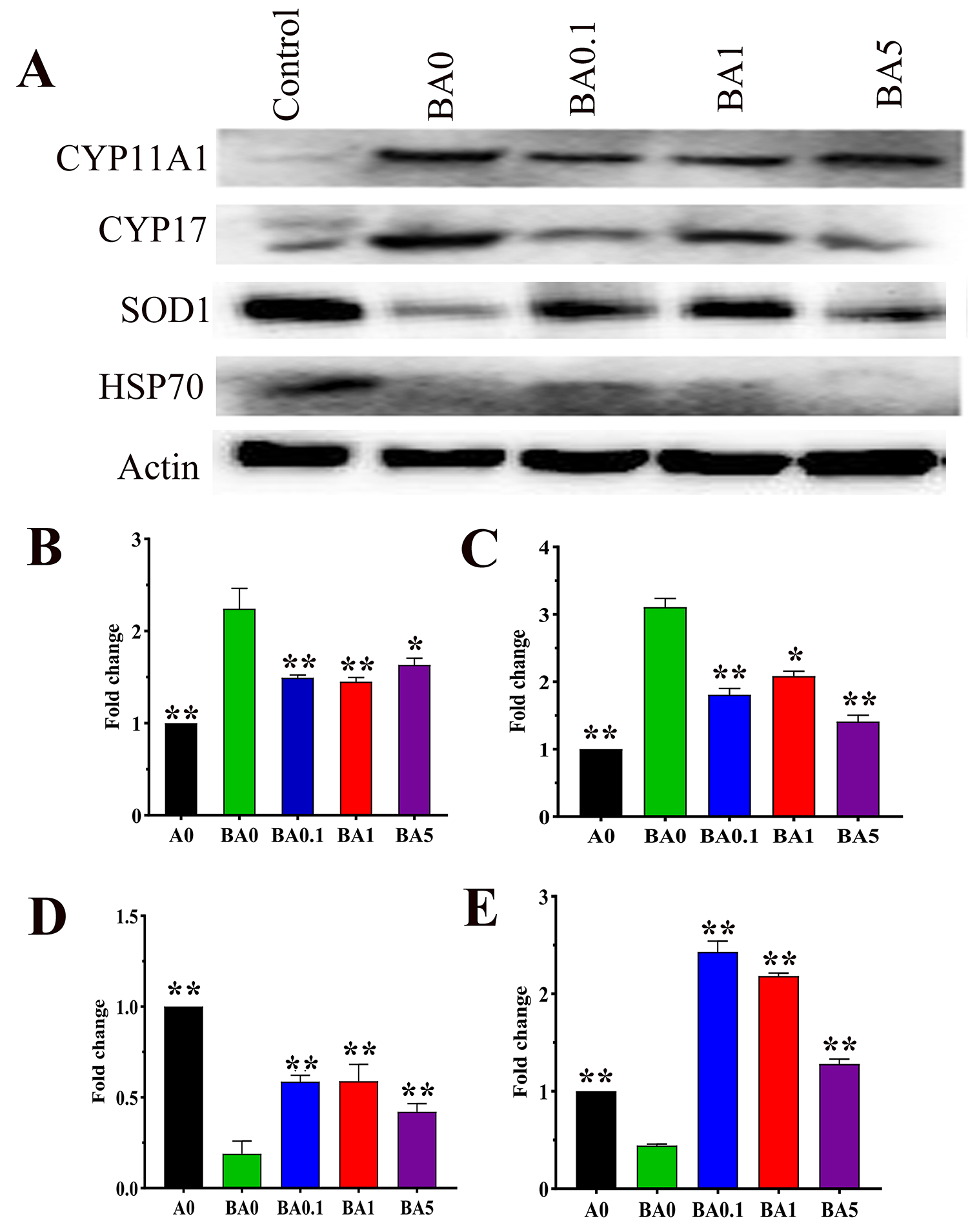

Supplement: Supplementary Figure S1 — (A) Protein levels of CYP11A1, CYP17, SOD1, and HSP70 in mice testis tissues detected by Western blotting, (B) Protein expression levels of CYP11A1, (C) Protein expression levels of CYP17, (D) Protein expression levels of SOD1, (E) Protein expression levels of HSP70, Relative expression was calculated using target protein/Actin. *Means 0.01 < p < 0.05 and **means p < 0.01. [file Image_1.tif]

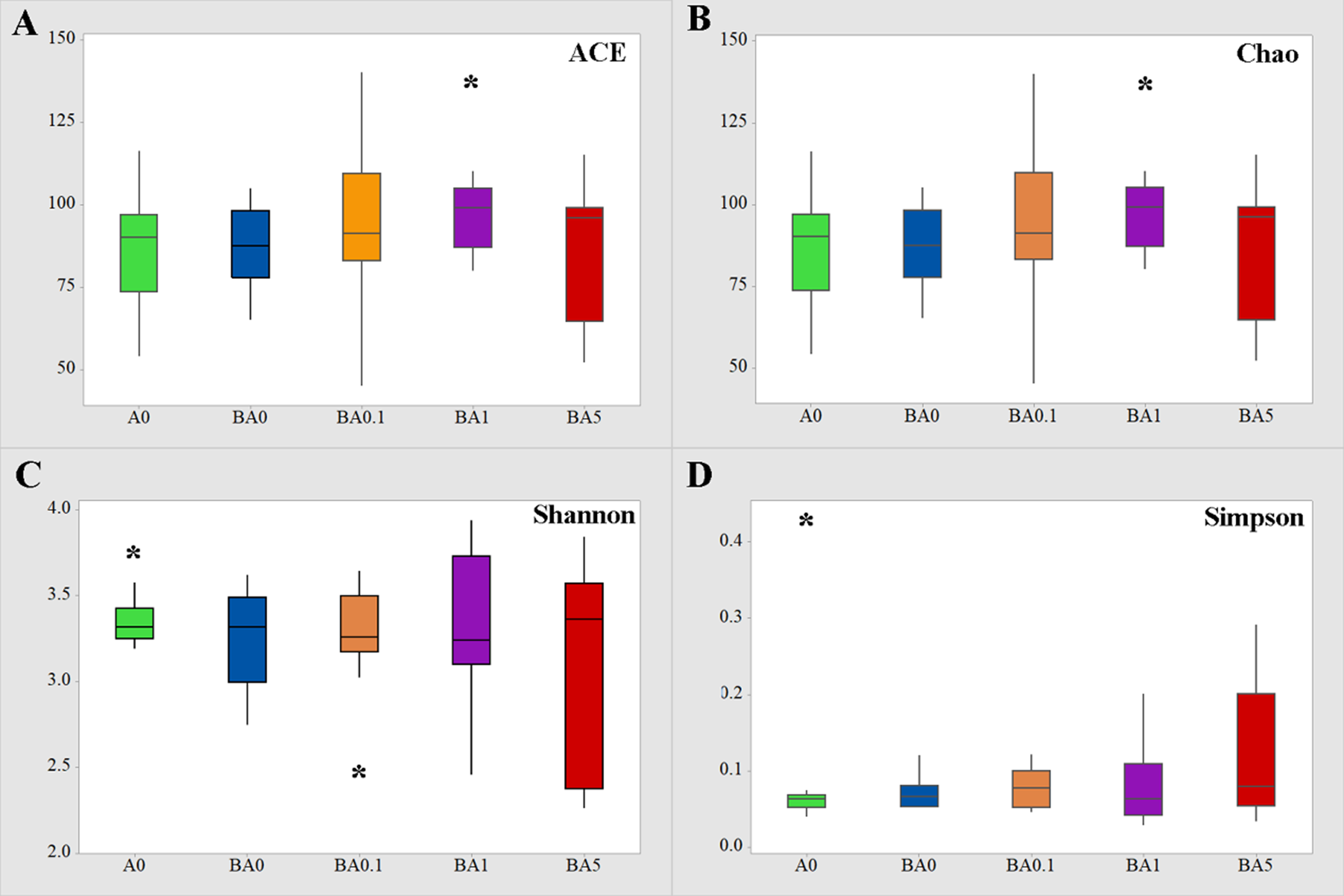

Supplement: Supplementary Figure S2 — The alpha index of the small intestine microbiota: (A) ACE index, (B) Chao index, (C) Shannon index, (D) Simpson index (n > 8 sample/group). [file Image_2.TIF]
